# Mediolateral Differences of Proteoglycans Distribution at the ACL Tibial Footprint: Experimental Study of 16 Cadaveric Knees

**DOI:** 10.1155/2018/3762580

**Published:** 2018-04-08

**Authors:** Joon Ho Wang, Byung Hoon Lee

**Affiliations:** ^1^Department of Orthopaedic Surgery, Samsung Medical Center, Sungkyunkwan University School of Medicine, Seoul 06351, Republic of Korea; ^2^Department of Health Sciences and Technology and Department of Medical Device Management and Research, SAIHST, Sungkyunkwan University, Seoul 06351, Republic of Korea; ^3^Department of Orthopaedic Surgery, Kang-Dong Sacred Heart Hospital, Hallym University Medical Center, Seoul, Republic of Korea

## Abstract

This study aimed to identify the staining pattern of ACL attachment blended with cartilage of the medial tibial plateau at the tibial insertion and histologically characterize the tibial footprint. Sixteen fresh frozen cadaveric knees (mean age: 52.0 ± 6.2 years) were used for this study. The specimens were bisected in the coronal plane, in accordance with the fiber orientation of the ACL tibial attachment. Adjacent sections were then stained with hematoxylin and eosin (H&E) to observe the morphology of the ACL insertion and with fast green and Safranin-O protocols to evaluate for collagen and proteoglycans (PG). The insertion area on the tibial footprint was divided into five zones in the medial to lateral direction, which was determined by division of the section from most prominent medial tibial spine to most lateral margin of ACL attachment. Then rectangular area with a vertical length that is twice the width of respective five zones was set. Stained areas of all images were quantified positively by using ImageJ software, and the value for staining area measured was defined in percentage by multiplying whole image area by 100. The mean proportion of Safranin-O staining is significantly greater nearer to the medial tibial spine (59% in zone 1, 32% in zone 2, 13% in zone 3, 13% in zone 4, and 4% in zone 5, *P* < 0.001). The medial section of the tibial insertion area grew in size and increased in PG staining with more densely organized collagen arrangement with more fibrocartilage cells. The ACL tibial insertion showed a medially eccentric staining pattern by histological evaluation of the ACL attachment to cartilage. Our histological results of the eccentric biomaterial property in the medial tibial spine of ACL insertion area can be considered in making a more functional anatomic tibial tunnel placement.

## 1. Introduction

Recent trends in anterior cruciate ligament (ACL) reconstruction have been oriented toward anatomic ACL reconstruction that restores the native size and location of the ACL insertion. Anatomic studies have been performed to understand the location of the ACL insertion [1–4]. The position of the tibial insertion site is bordered by Parsons' knob, the intercondylar ridge, the anterior horn of the lateral meniscus, and the medial and lateral tubercles [5]. A recent systematic review of basic science studies suggested that the anatomic centrum of the ACL tibial footprint lies 15 mm anterior to the posterior cruciate ligament and two-fifths of the M-L width of the interspinous distance [6]. Optimal anatomic replacement of the ACL graft is essential to improve patient clinical outcomes, and detailed knowledge of native insertions is critical [5].

However, anatomical reconstruction of the ACL does not restore the native insertion site in its entirety. There is no defined consensus of the restoration threshold that is needed to predict a successful or a poor outcome [7]. Accordingly, based on these reports, anteromedial placement of the tibial tunnel within anatomic footprints has been suggested for postoperative knee stability [8]. To restore effectively the function of the ACL after tearing, it is important to understand the native anatomical properties of the footprint.

Previous studies have described the mechanical properties and morphological analysis of the meniscus or ACL femoral insertion area [9] using histological examination and analysis. Tendons and ligaments have dynamic characteristics, and changes in mechanical load influence their response to the composition of the extracellular matrix (ECM) [10]. Therefore, histological evaluation of the ACL attachment to cartilage might give a reference to investigate the native mechanical properties of the entity of ACL footprint. This study was performed under the prerequisites that different mechanical properties can change the composition of the extracellular matrix (ECM) on the ACL attachment to cartilage even within the same ligamentous bundle. The purpose of this study is to identify the native anatomical properties of the ACL tibial footprint from histological examination of the staining pattern of ACL attachment to cartilage. We hypothesized that tibial tunnel for ACL reconstruction could be positioned under the consideration of the biomaterial property at the ligament insertion area.

## 2. Materials and Methods

**Figure 1:**
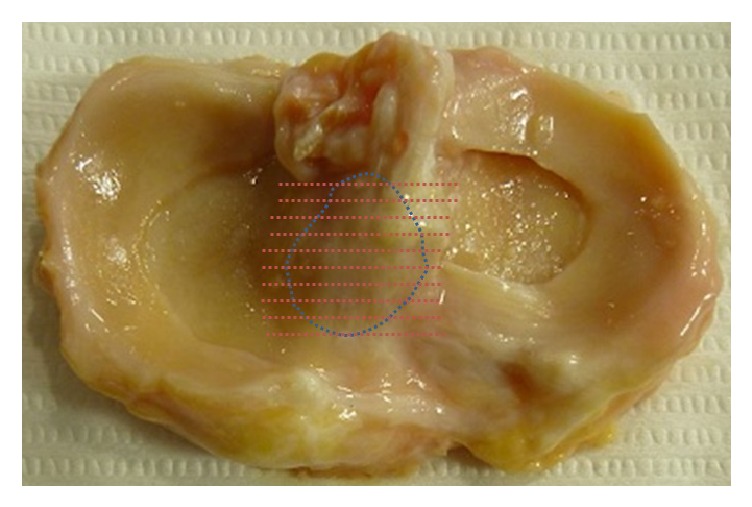
All specimens were bisected in the coronal plane, in accordance with fiber orientation of ACL tibial footprint (blue-dotted outline: ACL tibial footprint boundary; red-dotted lines: bisecting plane).

**Figure 2:**
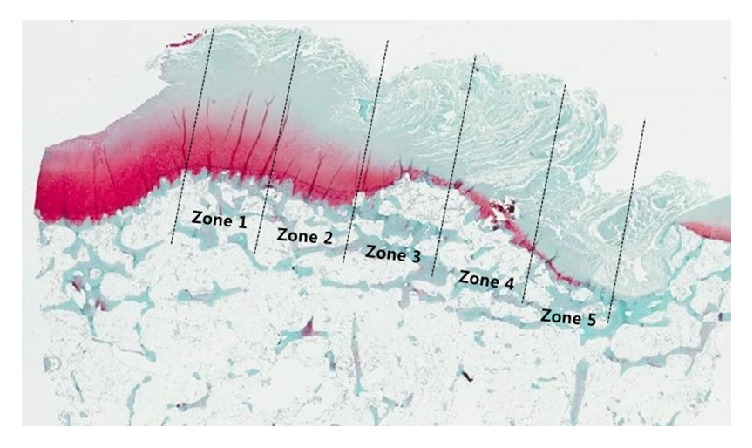
Microscopic measurements: divided into 5 zones to medial-lateral direction in direct insertion from medial tibial spine to border of ACL tibial insertion area.

**Figure 3:**
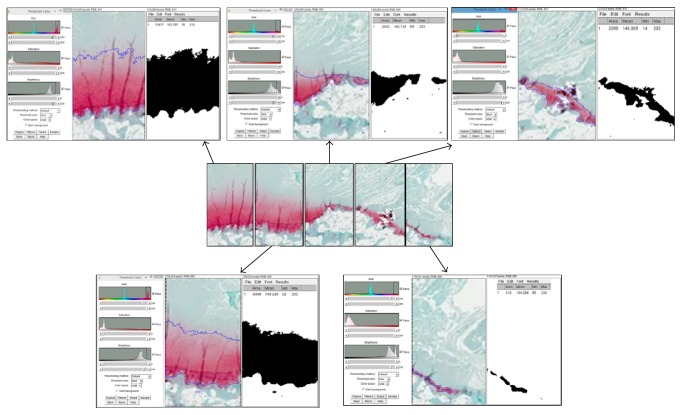
All images of each section segment can be segmented within targeted threshold color and the segmented image digitized; the black area can be calculated by using ImageJ software.

Eighteen fresh frozen cadaveric knees, removed from cadavers after use in our anatomy laboratory, were used for the evaluation of the tibia-ACL insertion. Two ACL-deficient, severely osteoarthritic knees were excluded from this study. The 16 remaining knees (from 8 males and 8 females) were included. The mean age of the subjects was 52.0 ± 6.2 years (range: 36 to 68 years). Each specimen was transected at mid-femur and mid-tibia. All soft-tissue structures around the knee were removed to expose the joint. The proximal tibia was cut by using an oscillating saw 2 cm below the articular surface to prevent damage to the ACL insertion, leaving intact the superficial fibrous membrane covering the ACL and ACL insertion. The morphology and position of the tibial ACL insertion were observed, while the ACL was moved with great care. Decalcification was performed using 30% formic acid at room temperature, depending on bone quality. The specimens were bisected in the coronal plane, embedded in paraffin, and sectioned (5 *μ*m thick) (Figure 1). Adjacent sections were then stained with hematoxylin and eosin (H&E) to observe the morphology of the ACL insertion and with fast green and Safranin-O protocols to evaluate for collagen and proteoglycans (PG). Among acquired slides, the one which included the highest portion of medial tibial spine was selected. Each specimen was carefully inspected with a light microscope, and the insertion area on the tibial footprint was divided into five zones in the mediolateral direction to compare the staining area (Figure 2). Five zones were determined by division of the section from most prominent medial tibial spine to most lateral margin of ACL attachment. All positively stained areas were quantified using image deconvolution methods as previously described (ImageJ; National Institutes of Health) (Figure 3) [11]. To medial to lateral direction, five zones were determined by division of the section from most prominent medial tibial spine to most lateral margin of ACL attachment. Then rectangular area with a vertical length that is twice the width of respective five zones was set. Stained areas of all images were quantified positively by using ImageJ software, and the value for staining area measured was defined in percentage by multiplying whole image area by 100.

*ImageJ Analysis.* Tagged image file format (TIFF) images were captured by Nikon E600 microscope at 20x magnification (Nikon Corporation, Melville NY, USA). Histological assessment of Safranin-O stain was performed. The TIFF image opened with ImageJ was adjusted to color threshold from hue, saturation, and brightness (HSB) to red, green, and blue (RGB) color space (Figure 3). In addition, color threshold was selected on red. The color threshold was adjusted for each image to depict the areas that display the positive areas of Safranin-O by defining minimum and maximum values for each color. The mask created by the color threshold was then changed from red to black and white (B&W). The image was changed to grayscale by setting image type to 8-bit. After converting the image to grayscale, the image was adjusted to threshold into B&W. To calculate total stained section area, the tool of “Analyze” was utilized and “Measure” was chosen. The value for total section area was recorded. The value obtained was defined in percentage by multiplying whole image area by 100. Ethical approval for this study was obtained from the ethical committee of our institution.

## 3. Statistical Analysis

All data are presented as means ± standard deviations with 25% to 75% quartile ranges. A priori power analysis was performed to determine the sample size using the two-sided hypothesis test at an *α* level of 0.05 and a power of 0.8. A post hoc power analysis was performed to determine whether the results of our study of 15 cases indicated adequate power, and the power was 91.1%, which was adequate to detect a 10% difference of staining area of each zone. Histological scoring was performed on two separate occasions by two separate researchers who were blinded to the results. They measured the stained areas twice, at each of the five sections of ACL tibial attachment of all knees, with an interval of 2 weeks between examinations. Reliability of the measurements was assessed by evaluating the intra- and intertester reliability using the intraclass correlation coefficient. Data from each experimental group were analyzed with a general linear model using One-Way ANOVA for repeated-measures procedures (and Bonferroni post hoc test). The 2-sample *t*-test was applied to compare the staining area (%) between two adjacent zones. Statistical significance was set as *P* < 0.05.

## 4. Results

**Figure 4:**
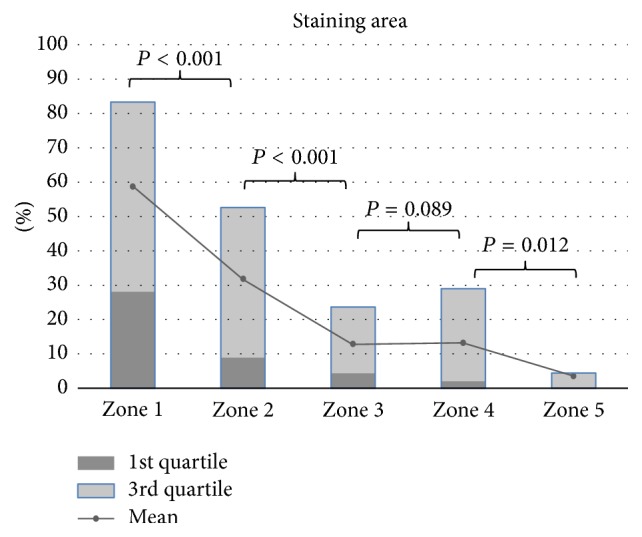
Graph of the Safranin-O staining pattern in ACL tibial footprint.

**Figure 5:**
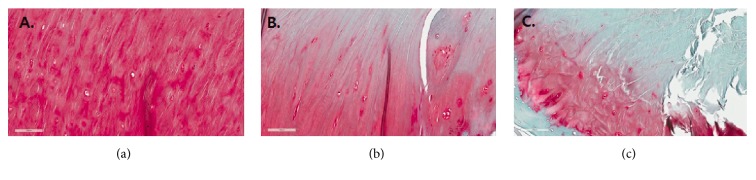
Staining pattern and collagen organization of three different zones (1, 3, and 5) in high power field view. (a) Note the strong staining pattern to PG and cuboid basketweave arrangement of collagen fibres with densely packed round shaped fibrocartilage cell (FC). (b) Zone 3 shows the staining pattern of longitudinal collagen organization parallel to fiber orientation with fewer FC and lower peak color of staining compared to zone 1. (c) Zone 5 shows the staining pattern of scarce interspersed FC and disorganized collagen orientation with staining only at the adjacent area of the tibial footprint.

The staining pattern at the ACL tibial attachment to cartilage indicated that ligament roots may continue into the articular cartilage, where they then blend with cartilage nearer to the medial tibial spine (Figure 2). The proportion of Safranin-O staining is significantly greater nearer to the medial tibial spine. This staining pattern is evident in the medial section of the tibial insertion area. The mean proportion of Safranin-O staining is significantly greater nearer to the medial tibial spine (59% in zone 1, 32% in zone 2, 13% in zone 3, 13% in zone 4, and 4% in zone 5, *P* < 0.001) (Table 1 and Figure 4). Medial portion of the tibial insertion area grew in size and increased in PG staining with more densely organized collagen arrangement and more fibrocartilage cells seen under high magnification (Figure 5). The inter- and intratester reliability ranged from 0.79 to 0.90 and from 0.81 to 0.92, respectively (more information can be seen at an appendix as a supplemental file, Table s1).

## 5. Discussion

The principal finding of this study is that ACL insertion has a ligament-like structure with increased PG content near to the medial tibial spine. Even though it would not be definite that eccentric staining pattern in medial to lateral direction is resultant in histological results by overlapping between ligament insertion area and medial tibial cartilage or transitions into the fibrocartilaginous structure near the medial tibial spine by eccentric load, the result indicates a discrete discrepancy of biomaterial property of ACL tibial insertion area.

A deep understanding of the basic science and anatomy is the key to evaluating the pathologic and morphologic changes in the ACL and its insertion site [12]. In ACL femoral footprint, direct insertion area showed the structural transition from the collagenous ligament bundle into the fibrocartilaginous attachment [4]. It allows for the transmission of the complex loading of the ACL fibers into the femoral insertion.

This is the first study of the structural transition between the insertional ligaments (footprint) and the cartilaginous body of the ACL tibial footprint. The medial portion of ACL tibial insertion with increase in PG content would be developed by prestress in the structure through osmotic swelling [10]. This structure of the medial portion of ACL tibial insertion results in the much greater shear and compressive stiffness than the lateral portion, as evidenced by its resistance to changes in shape, which might be more resistant to shear forces induced by the interaction with the adjacent cruciate ligaments and articular cartilage.

There have been several references regarding the real anatomic tibial footprint. Ferretti et al. [13] reported on 2 bony prominences that exist in the anterior edge and partition the anteromedial and posterolateral bundle in the femoral ACL footprint; these bony protrusions were coined the “intercondylar ridge” and “bifurcate ridge,” respectively. The CT study reporting on tibial bony landmarks by Purnell et al. [14] is widely known. The authors reported that the posterior and medial boundaries of bone are the tibial ridge and medial intercondylar eminence, respectively; there is no bony landmark in the anterior and lateral boundaries. However, compared with the femoral attachment, there are no general standard guidelines, and there are fewer reports on the bony landmarks for anatomic placement of the tibial tunnel.

ACL femoral insertion area can be defined by bony ridges identified after removing all the remnant tissues. Meanwhile, it is difficult to define the accurate tibial tunnel position because (1) it is quite limited to remove the remnant tissue on the tibial attachment for identification of the accurate boundary of tibial footprint due to possible injury of anterior horn of meniscus blended to distal fibers of ACL; (2) removing the distal remnant for identification of the accurate boundary of tibial footprint can lower the healing potential, in which blood supply is concentrated to ligament; (3) it is difficult to create an ideal tibial tunnel compatible with the diverse shape of the tibial footprint; and (4) grafts have a limited coverage of anatomic footprint in its entirety.

The limited coverage of the anatomic footprint by grafts suggests the importance of searching for a more functional position within the tibial footprint. Recent studies showed the importance of the tibial tunnel location in terms of translational control and rotational stability after ACL reconstruction [15–19]. Bedi et al. [16], from results in a cadaveric study, reported that a more anterior tibial tunnel placement significantly reduced anterior tibial translation and pivot-shift movement compared with posterior tunnel. Mall et al. [19] revealed that less oblique grafts were associated with greater anterior translation and that graft obliquity was particularly influenced by tibial tunnel position. Accordingly, based on these reports, it is important for postoperative knee stability to determine anteromedial placement of the tibial tunnel within anatomic footprints.

Our histological results are especially important to establish tibial tunnel position in the reconstruction. We speculated that eccentric biomaterial property to medial tibial spine of ACL insertion area would suggest a medially positioned tibial tunnel. Consideration of anatomic discrepancies with scientific rationale can be applicable to obtaining a more functional enhancement.

This study has several limitations. First, the observation of the material property was performed only in the coronal plane. Second, we did not present the scientific basis of whether the actual mechanical property is relevant to the histological finding. Third, morphological assessment of the cartilage at the ACL tibial footprint was presented only using Safranin-O staining. However, cartilage isolated from cartilage and meniscus from the joint was proven to show the greatest intensity of staining for type I collagen, with weak staining for other collagens such as types V, VI and X [20]. Therefore, the findings using Safranin-O staining can be claimed for the morphological assessment of the cartilage. Fourth, ligament property can be changed with cartilage metamorphosis by degenerative change. However, we could find visually intact ACL fibers with no degeneration except two specimens excluded in thus study.

## 6. Conclusions

The ACL tibial insertion showed a medially eccentric staining pattern by histological evaluation of the ACL attachment to cartilage. Our histological results of the eccentric biomaterial property in the medial tibial spine of ACL insertion area can be considered in making a more functional anatomic tibial tunnel placement.

## Figures and Tables

**Table 1:** Staining area for each of the five sections at the tibial attachment area of AM and PL bundle.

(*N* = 16)	Area ± SD (25–75%)					*P* ^*∗*^
	Zone 1	Zone 2	Zone 3	Zone 4	Zone 5	
Staining	58.8 ± 29.1	31.8 ± 24.8	12.9 ± 13.1	13.2 ± 15.7	3.6 ± 5.8	**<0.001**
Area (%)	(28.2–83.3)	(8.9–52.6)	(4.4–23.6)	(2.0–28.9)	(0–4.5)	
*P* ^†^	**<0.001**	**<0.001**	**<0.001**	0.089	**0.012**	
			0.089	**0.012**		

^*∗*^Differences among the staining area (%) of five zones were evaluated using analysis of variance (ANOVA) model. ^†^Comparison of the staining area (%) between two adjacent zones. ^*∗*†^Values of *P* < 0.05 are displayed in bold.

## Data Availability

The datasets used and/or analyzed during the current study are available from the corresponding author on reasonable request.

## Supplementary Materials

Supplementary Materials Table s1: results of intraclass correlation coefficient (ICC) value of each measurement.

## References

[1] Girgis F. G., Marshall J. L., Al Monajem A. R. S. (1975). The cruciate ligaments of the knee joint: anatomical, functional and experimental analysis. *Clinical Orthopaedics and Related Research*.

[2] Amis A. A., Dawkins G. P. (1991). Functional anatomy of the anterior cruciate ligament. Fibre bundle actions related to ligament replacements and injuries. *The Journal of Bone & Joint Surgery (British Volume)*.

[3] Colombet P., Robinson J., Christel P. (2006). Morphology of anterior cruciate ligament attachments for anatomic reconstruction: a cadaveric dissection and radiographic study. *Arthroscopy: The Journal of Arthroscopic and Related Surgery*.

[4] Iwahashi T., Shino K., Nakata. K., Otsubo H., Suzuki T., Amano H. (2010). Direct anterior cruciate ligament insertion to the femur assessed by histology and 3-dimensional volume-rendered computed tomography. *Arthroscopy: The Journal of Arthroscopic & Related Surgery: Official Publication of The Arthroscopy Association of North America and The International Arthroscopy Association*.

[5] Tensho K., Shimodaira H., Aoki T. (2014). Bony landmarks of the anterior cruciate ligament tibial footprint: A detailed analysis comparing 3-dimensional computed tomography images to visual and histological evaluations. *The American Journal of Sports Medicine*.

[6] Schillhammer C. K., Reid J. B., Rister J. (2016). Arthroscopy Up to Date: Anterior Cruciate Ligament Anatomy. *Arthroscopy : the journal of arthroscopic & related surgery : official publication of the Arthroscopy Association of North America and the International Arthroscopy Association*.

[7] Middleton K. K., Muller B., Araujo P. H. (2015). Is the native ACL insertion site “completely restored” using an individualized approach to single-bundle ACL-R?. *Knee Surgery, Sports Traumatology, Arthroscopy*.

[8] van Eck C. F., Lesniak B. P., Schreiber V. M., Fu F. H. (2010). Anatomic single- and double-bundle anterior cruciate ligament reconstruction flowchart. *Arthroscopy: The Journal of Arthroscopic & Related Surgery: Official Publication of The Arthroscopy Association of North America And The International Arthroscopy Association*.

[9] Sasaki N., Ishibashi Y., Tsuda E. (2012). The femoral insertion of the anterior cruciate ligament: Discrepancy between macroscopic and histological observations. *Arthroscopy - Journal of Arthroscopic and Related Surgery*.

[10] Benjamin M., Ralphs J. R. (1998). Fibrocartilage in tendons and ligaments - An adaptation to compressive load. *Journal of Anatomy*.

[11] Varghese F., Bukhari A. B., Malhotra R., De A. (2014). IHC profiler: an open source plugin for the quantitative evaluation and automated scoring of immunohistochemistry images of human tissue samples. *PLoS ONE*.

[12] Oka S., Schuhmacher P., Brehmer A., Traut U., Kirsch J., Siebold R. (2016). Histological analysis of the tibial anterior cruciate ligament insertion. *Knee Surgery, Sports Traumatology, Arthroscopy*.

[13] Ferretti M., Ekdahl M., Shen W., Fu F. H. (2007). Osseous Landmarks of the Femoral Attachment of the Anterior Cruciate Ligament: An Anatomic Study. *Arthroscopy - Journal of Arthroscopic and Related Surgery*.

[14] Purnell M. L., Larson A. I., Clancy W. (2008). Anterior cruciate ligament insertions on the tibia and femur and their relationships to critical bony landmarks using high-resolution volume-rendering computed tomography. *The American Journal of Sports Medicine*.

[15] Ayerza M. A., Múscolo D. L., Costa-Paz M., Makino A., Rondón L. (2003). Comparison of sagittal obliquity of the reconstructed anterior cruciate ligament with native anterior cruciate ligament using magnetic resonance imaging. *Arthroscopy - Journal of Arthroscopic and Related Surgery*.

[16] Bedi A., Maak T., Musahl V. (2011). Effect of tibial tunnel position on stability of the knee after anterior cruciate ligament reconstruction: Is the tibial tunnel position most important?. *The American Journal of Sports Medicine*.

[17] Hatayama K., Terauchi M., Saito K., Higuchi H., Yanagisawa S., Takagishi K. (2013). The importance of tibial tunnel placement in anatomic double-bundle anterior cruciate ligament reconstruction. *Arthroscopy - Journal of Arthroscopic and Related Surgery*.

[18] Inderhaug E., Strand T., Fischer-Bredenbeck C., Solheim E. (2014). Effect of a too posterior placement of the tibial tunnel on the outcome 10-12 years after anterior cruciate ligament reconstruction using the 70-degree tibial guide. *Knee Surgery, Sports Traumatology, Arthroscopy*.

[19] Mall N. A., Matava M. J., Wright R. W., Brophy R. H. (2012). Relation between anterior cruciate ligament graft obliquity and knee laxity in elite athletes at the national football league combine. *Arthroscopy - Journal of Arthroscopic and Related Surgery*.

[20] Naumann A., Dennis J. E., Awadallah A. (2002). Immunochemical and mechanical characterization of cartilage subtypes in rabbit. *Journal of Histochemistry & Cytochemistry*.

