# Supplementary material for: Mediolateral Differences of Proteoglycans Distribution at the ACL Tibial Footprint: Experimental Study of 16 Cadaveric Knees
Source: Biomed Res Int. 2018 Apr 8;2018:3762580. doi: 10.1155/2018/3762580 (PMC5911328; doi:10.1155/2018/3762580)
Supplement: Supplementary Materials — Table s1: results of intraclass correlation coefficient (ICC) value of each measurement. [file 3762580.f1.docx]

**Table s1.** Results of Intraclass Correlation Coefficient (ICC) Value of Each Measurement.

|  | Stained Area of ACL tibial insertion | | | | |
| --- | --- | --- | --- | --- | --- |
|  | Zone 1 | Zone 2 | Zone 3 | Zone 4 | Zone 5 |
| Inter-tester |  |  |  |  |  |
| ICC | 0.85 | 0.82 | 0.79 | 0.90 | 0.86 |
| Lower ICC | 0.75 | 0.72 | 0.69 | 0.76 | 0.71 |
| Upper ICC | 0.95 | 0.96 | 0.90 | 0.94 | 0.91 |
| Intra-tester |  |  |  |  |  |
| ICC | 0.81 | 0.91 | 0.92 | 0.91 | 0.87 |
| Lower ICC | 0.75 | 0.85 | 0.84 | 0.86 | 0.80 |
| Upper ICC | 0.89 | 0.95 | 0.96 | 0.93 | 0.91 |
